# Analyzing BMP2, FGFR, and TGF Beta Expressions in High-Grade Osteosarcoma Untreated and Treated Autografts Using Proteomic Analysis

**DOI:** 10.3390/ijms23137409

**Published:** 2022-07-03

**Authors:** Rashmi Madda, Chao-Ming Chen, Cheng-Fong Chen, Jir-You Wang, Hsin-Yi Wu, Po-Kuei Wu, Wei-Ming Chen

**Affiliations:** 1 Orthopedic Department, School of Medicine, National Yang-Ming University, Taipei 112, Taiwan; drrashmimadda@gmail.com (R.M.); excelnova@gmail.com (C.-M.C.); cf_chen@vghtpe.gov.tw (C.-F.C.); yollywang@gmail.com (J.-Y.W.); wmchen@vghtpe.gov.tw (W.-M.C.); 2 Department of Orthopedics and Traumatology, Taipei Veterans General Hospital, Taipei 112, Taiwan; 3 Therapeutical and Research Center of Musculoskeletal Tumor, Taipei Veterans General Hospital, Taipei 112, Taiwan; 4 Pacific Northwest National Laboratory, Richland, WA 99354, USA; 5 Instrumentation Center, National Taipei University, Taipei 237, Taiwan; hsinyiwu@ntu.edu.tw

**Keywords:** biological reconstruction, target proteomics, bone morphogenetic proteins, mass spectrometry, growth factors, biological reconstruction, BMP2, TGF-Beta, osteosarcoma

## Abstract

In the last few decades, biological reconstruction techniques have improved greatly for treating high-grade osteosarcoma patients. To conserve the limb, and its function the affected tumor-bearing bones have been treated using liquid nitrogen and irradiation processes that enable the removal of entire tumors from the bone, and these treated autografts can be reconstructed for the patients. Here, we focus on the expressions of the growth factor family proteins from the untreated and treated autografts that play a crucial role in bone union, remodeling, and regeneration. In this proteomic study, we identify several important cytoskeletal, transcriptional, and growth factor family proteins that showed substantially low levels in untreated autografts. Interestingly, these protein expressions were elevated after treating the tumor-bearing bones using liquid nitrogen and irradiation. Therefore, from our preliminary findings, we chose to determine the expressions of BMP2, TGF-Beta, and FGFR proteins by the target proteomics approach. Using a newly recruited validation set, we successfully validate the expressions of the selected proteins. Furthermore, the increased growth factor protein expression after treatment with liquid nitrogen may contribute to bone regeneration healing, assist in faster recovery, and reduce local recurrence and metastatic spread in high-grade sarcoma patients.

## 1. Introduction

The most common malignant tumor that arises from the bone is osteosarcoma (OGS). This is commonly observed in children and the middle-aged population [1]. The histological investigations found that OGS typically arises from the mesenchymal stem cells and spindle cells that characterize osteoid formation [2]. Therefore, curative approaches such as surgery and chemotherapy were employed as treatment options that typically showed unsatisfactory results in patients [1]. The commonly known chemotherapy drugs include Doxorubicin, Methotrexate, Cisplatin, and other combinational drugs that are often accompanied by significant side effects [3,4]. As we all know is that bone is the third most common metastatic tumor after the lungs and liver.

Moreover, OGS primarily metastasizes to the lungs and liver and spreads to the other major organs [1]. On the other hand, most metastatic patients develop severe pain, and it is often difficult to achieve pain relief, resulting in poor quality of life and losing their organs [5]. Furthermore, organ donors are not readily available, and matching the same organ has proven to be predominantly difficult. Therefore, physicians choose an alternative treatment option such as biological reconstruction techniques [6] that use ablative procedures such as freezing or irradiation to kill the tumor from the affected bone and reuse the same bone (autografts) to reconstruct the bone defects after the tumor resection. This technique uses liquid nitrogen (LN), a lethal temperature cryogen, to augment tissue necrosis by forming ice crystals, resulting in the dehydration of cells and killing tumor cells effectively [7]. It helps in the revitalization of the bone to achieve union and subsequent remodeling. A soft tissue attachment to the bones can also be restored, resulting in greater efficiency and functionality of the limbs. In recent times, freezing has been clinically proven and applied to various tumors, including skin, breast, lungs, kidneys, liver, prostate, esophagus, and bones [8]. Furthermore, several studies have demonstrated that freezing is a safe, efficient, and durable palliation method that reduces pain, metastatic potential, and recurrence [8,9]. In addition to this, our previous clinical study that was conducted on 164 high-grade OGS patients who received autografts (79 received irradiation-treated, and 82 received freezing-treated) showed excellent treatment response in revitalizing the bone union by achieving significant remodeling [10]. Further, we have also observed faster recovery, less metastasis, and recurrence rates with no complication rates. To recover and heal faster and achieve better bone union, growth factors are crucial factors [11]. A recent study by Takata et al. verified an important growth factor, which is bone morphogenetic protein (BMP)-7 expressions among LN freezing and pasteurization revealed, and that freezing preserved better bioactivity than an allograft, which possibly shows better potential for graft survival [12]. In a later study by our group, Chen et al. showed that bone morphogenic proteins (BMP2 and BMP7) preserved better bioactivity in LN-freezing and irradiation treated autografts than in non-treated ones [13]. In addition, a recent study by Xu et al. demonstrated that rabbit pedicle freezing autografts showed higher survival rates when expressing several bone revitalization proteins [14]. Recently, we used proteomics to explore and study the freezing and irradiation treated autografts of osteosarcoma, chondrosarcoma, and giant cell tumor in greater detail [15,16]. Our previous proteomic investigations uncovered several proteins whose expressions were positively altered and may be able to restore and remodel bone defects through regulating several pathways. This led us to focus on the growth factor proteins and how they respond to freezing and irradiation. These proteins are vital for the homeostasis of various tissues, including bone, cartilage development, revitalization, repair, and healing. Based on our extensive literature search, no previous study compared the protein expression changes of LN-freezing treated and irradiation treated autografts compared to untreated using a targeted proteomic approach. The rapidly advancing proteomic technology, along with mass spectrometry, is a highly sensitive and powerful tool that allows us to explore large data sets to quantify and find the potential protein targets from any given biological sample. The target proteomic assay is a high-throughput, quantitative, and statistically robust technique that verifies our proteins of interest without using expensive antibodies. Furthermore, the most significant advantage of using target proteomic technology is the ability to multiplex a large number of samples and highly reproducible results.

## 2. Results

### 2.1. Sample Processing Information

**Figure 1:**
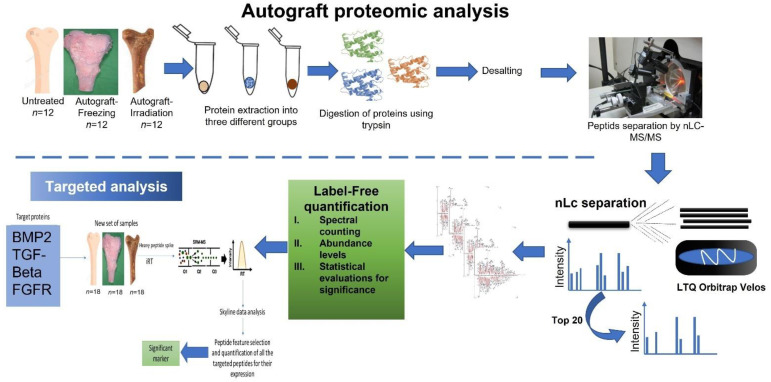
High-grade osteosarcoma untreated, freezing-treated, and irradiation-treated autografts target proteomic analysis workflow.

For the proteomic analysis, freshly collected osteosarcoma femoral heads (n = 48) were obtained during the biological reconstruction surgery. Informed consent was obtained from each applicant, and this study was approved by the Institutional Review Board of Taipei Veterans General Hospital. The collected samples were grouped as non-treated (n = 16), LN-freezing treated (n = 16), and irradiation treated (n = 16). For the freezing-treated group, femoral heads were treated using liquid nitrogen for 15 min, and for the irradiation-treated group, the sample was exposed to 15,000 gamma irradiations. After the treatments, all the bone samples were stored and −80 °C for further analysis. The complete workflow of our proteomic analysis is illustrated in Figure 1.

### 2.2. Differential Protein Expressions among LN-Freezing Treated and Irradiation-Treated Autografts with Untreated Autografts

**Figure 2:**
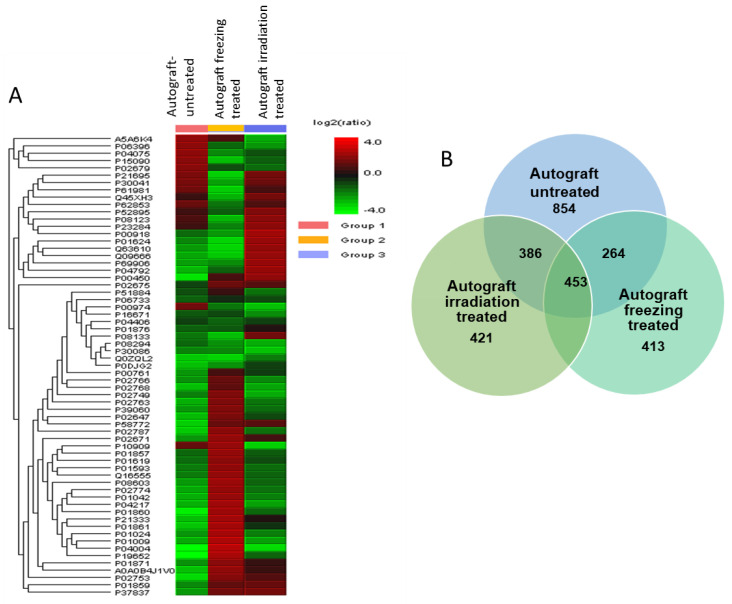
(**A**). Heatmap showing the protein expression changes among the treated and untreated autografts, (**B**). Ven diagram illustrates the identified proteins in individual groups and the overlapped proteins among them.

Samples from three groups were extracted and digested using trypsin and analyzed by LC-ES-MS/MS analysis. A total of 1688 proteins were consistently identified from three groups. To obtain the differential expressions among three groups, PEAKS X software (Bioinformatics Solutions Inc., Waterloo, ON, USA) was used to quantify the results and find the altered expressions of proteins. Each group has identified several potential proteins that are involved in various regulating pathways. The autograft-untreated was identified with 854 proteins, the freezing-treated was identified with 413, and the irradiation-treated was identified with 421 proteins. The similarly expressed proteins among the three groups were 453 and the commonly identified proteins among autograft-untreated and freezing-treated were 264. On the other hand, the irradiation-treated compared to untreated autografts had 386 proteins similarly identified. To understand the identified proteins and their expressions in each group, all the peptide intensities and their significance levels were quantified, and the abundance levels were compared using PEAKS X software with a stringent filtering criterion. (Figure 2A,B).

The heatmap from Figure 2A illustrates some proteins were highly expressed and the others were poorly expressed in untreated autografts. While the treatment groups showed there were significant protein expression changes that occurred after the treatment. We filtered the differentially expressed proteins from our study by using the false discovery rate (FDR) of 0.1%, the highest protein score of >70 with a significance score of <20, and the identification of at least two up to ten unique peptides. Accordingly, we were able to identify 78 proteins that were significantly expressed in autograft untreated compared to treated. The ratio of proteins from each group was measured among triplicate samples, and the results were highly correlated across the three groups were correlated (r = 0.91, Supplementary Figure S1). 

**Figure 3:**
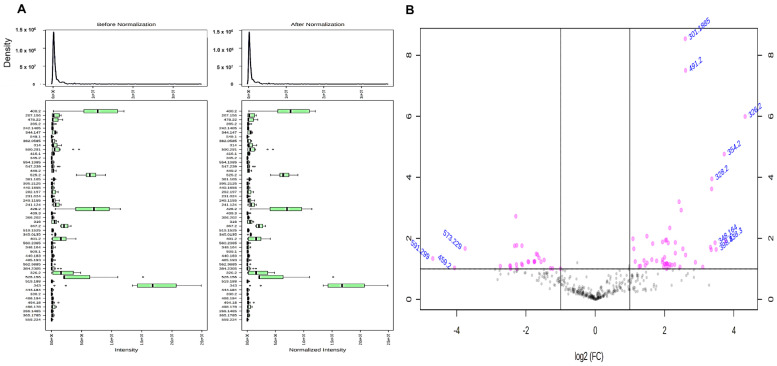
(**A**). A representative figure showing the identified proteins before and after normalization of proteins from three groups, (**B**). Volcano plot of the identified proteins and their differential expressions in log2 scale.

To obtain the accuracy of the quantitative data, the obtained results were normalized and the before and after normalization of the obtained data sets among autograft-untreated and autograft-freezing were shown in Figure 3A. Additionally, the proteins that are altered with higher and lower expressions were in the volcano plot of Figure 3B. The identified proteins were quantified using one-way ANOVA and the statistical analysis significance was measured from 0.01 to 0.05. Our quantification results revealed that among 78 significantly altered proteins, 18 proteins were up-regulated >3.0–1.5-fold (*p* < 0.05 or 0.01) in autograft-untreated and 60 proteins were down-regulated. On the other hand, 36 proteins showed higher >3.0–1.5-fold (*p* < 0.05 or 0.01) expressions in the freezing-treated group and 42 proteins were identified with down-regulated expressions <0.2–0.5-folds (*p* < 0.01 or 0.05). Then, the irradiation-treated group showed 25 up-regulated proteins >2.5–1.5-fold (*p* < 0.05 or 0.01) and 53 proteins with down-regulated expressions <0.1–0.5-folds (*p* < 0.01 or 0.05). All the identified protein groups and their expressions are listed in Supplementary Figure S1.

### 2.3. Gene Ontology Analysis and KEGG Pathway

**Figure 4:**
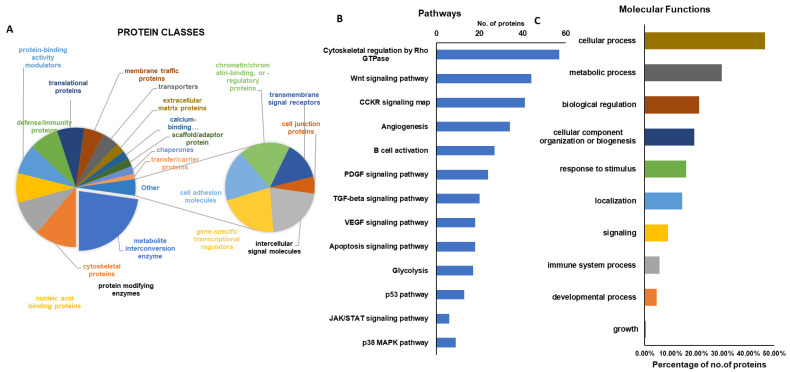
(**A**). Gene ontology analysis of identified proteins classifications, (**B**). Number of proteins from this study that are involved in various potential pathways, (**C**). Molecular functions of the identified proteins from the untreated and treated autografts.

The significantly altered higher expressions of proteins identified after freezing and irradiation treatment on autografts were further analyzed using functional enrichment analysis by GO-Terms. Most of the identified proteins in this study were classified into a wide range of biological, cellular, and metabolic processes, and their regulatory mechanisms. Figure 4A shows the various protein groups that are involved in crucial metabolic pathways may play a key role in the repair and regeneration of bone growth. Furthermore, these proteins are also implicated in various crucial pathways, including cytoskeletal regulation, TGF-beta signaling, VEGF signaling, apoptosis, angiogenesis, Wnt signaling, B-cell activation, and more, as can be seen in Figure 4B. As well some of the proteins were associated with the cellular component organization, developmental process, and growth were illustrated in Figure 4C. These findings demonstrate that ablative treatment regulates a wide range of cellular (50%) and metabolic (<30%), biological (<20%) processes, which aids in the preservation of bone activity, union, and regeneration.

### 2.4. Protein–Protein Interaction (PPI) Networks

**Figure 5:**
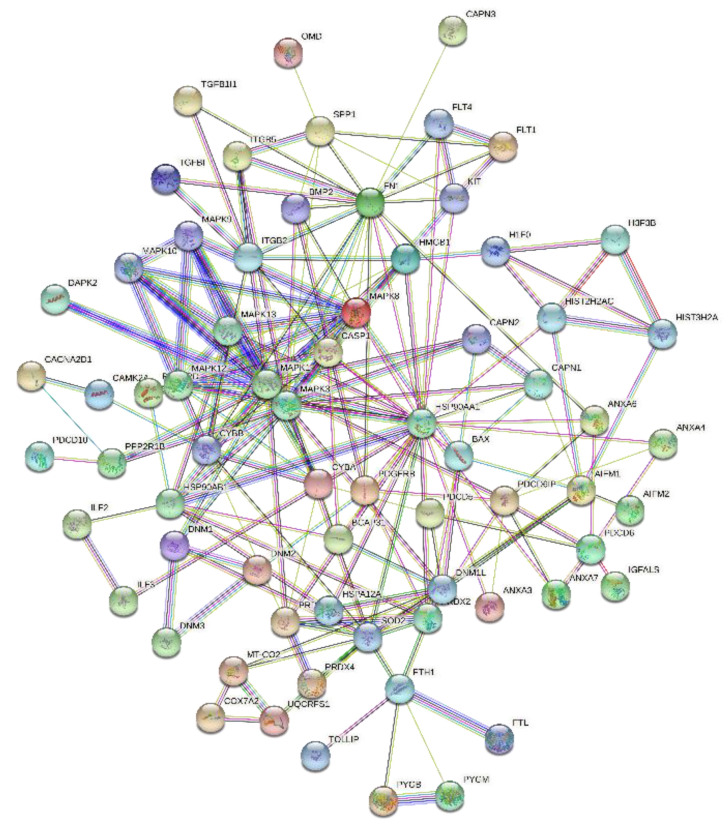
Protein–protein interaction (PPI) network from string analysis showing the tight network among growth factor proteins.

To understand the identified proteins and their interactions with each other, we employed PPI network analysis using STRING with the highest confidence score of 0.9. The PPI interactive network revealed that the majority of the proteins have tightly interacted and Figure 5 demonstrated the strong network among the growth factor proteins and metabolic proteins that are involved in the regulation of cellular proliferation and differentiation.

### 2.5. Bone Growth and Activity Preserving Protein Expressions Validation

**Figure 6:**
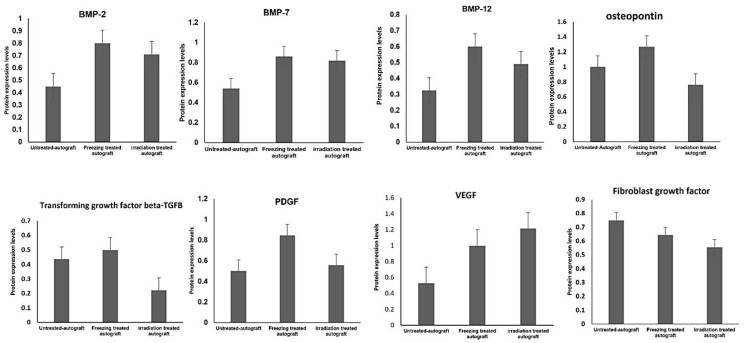
Protein abundance difference of the identified BMPs and growth factor proteins from the mass spectrometry analysis.

From our observations, we found some potential growth-related proteins such as bone morphogenic protein-2, bone morphogenic protein-7, TGF-beta, osteopontin, VEG, TGF-Beta, fibroblast growth factor (FGF), platelet-derived growth factor (PDGF), and MMP9 showed positive regulation after freezing and irradiation treatments. The goal of this study is to focus on the growth factor-related protein identifications and compare their expressions among the untreated and treated autografts. Interestingly, the above-mentioned growth factor proteins are found with altered expressions, and these proteins play an important role in bone regeneration, healing, growth, and recovery processes. Thus, we chose two platforms, such as target proteomics and Western blot analysis, to confirm BMP2, TGF-Beta, and MMP9 expressions using a newly recruited patient sample. From our mass spectrometry analysis, these proteins showed altered expressions, especially when we compared them with untreated autografts. Most of them were identified with reduced expressions as expected due to the tumor growth and it may diminish the expressions of these potential proteins shown in Figure 6.

**Figure 7:**
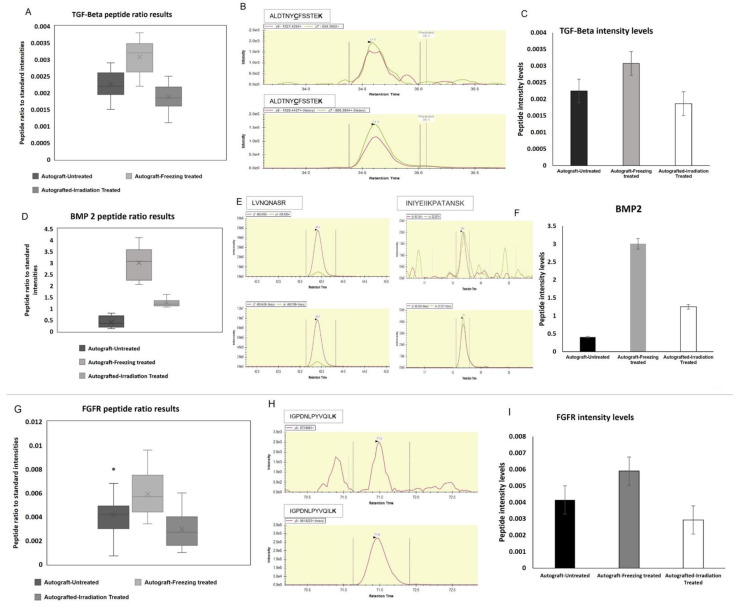
Three proteins (BMP2, TGF-Beta, and FGFR) were detected by target proteomic analysis. (**A**) TGF-Beta endogenous peptides detection to the heavy spiked standard ratios were illustrated (**B**) Detectability of TGF-Beta light to heavy peptides identification (**C**) Comparison of TGF-beta endogenous intensity levels among three groups of autografts. (**D**) BMP2 protein endogenous peptides detection to the heavy spiked standard ratios were illustrated (**E**) Detectability of BMP2 light to heavy peptides identification (**F**) Comparison of FGFR endogenous intensity levels among three groups of autografts (**G**) FGFR detection of endogenous peptides to the heavy spiked standard ratios were illustrated, the gray dot represents any data not included between the whiskers is an outlier (**H**) Detectability of FGFR light to heavy peptides (**I**) Comparison of FGFR endogenous intensity levels among three groups of autografts.

The interesting finding is that after freezing and irradiation treatment of autografts, these growth factor proteins such as the BMP2, BMP7, TGF-Beta, PDGF, and VEGF expressions were tremendously increased. The increased expressions of growth factors after the treatment demonstrated the treatment effect on the tumor-bearing bone, and it 100% killed the tumor and restored the crucial proteins and their functions, which are important for bone repair, healing, and restoring. Therefore, we chose three proteins such as BMP2, TGF-Beta, and MMP9 to confirm the expression levels using target proteomic analysis and Western blotting techniques using another newly recruited set of high-grade sarcoma patients (n = 18) of untreated and treated (n = 18) for confirmation analysis. Based on the verification study, we confirmed that the mass spectrometric analysis was significantly related to the consistent expression patterns of BMP2, TGF-beta, and MMP9 (*p* < 0.001). Figure 7A showed the target protein expressions of heavy and endogenous (light) peptide ratios, and the heavy and light peptide detection and peak area expression were illustrated in Figure 7B. To understand the correlation between mass spectrometry and validation data, Figure 7C showed the abundance levels of the selected proteins.

### 2.6. Target Proteins Validation

We chose three potential target proteins such as TFGF-Beta, BMP2, and FGFR to validate their differential expressions using selection reaction monitoring (SRM) mass spectrometry methodology using a newly recruited sample set of high-grade sarcoma patients. The SRM assay was developed based on the prior analysis. We chose the retention time and the transitions of the target peptides from untreated and treated high-grade sarcoma autografts (a total of 44 samples). TGF-Beta, BMP2, and FGFR were successfully quantified and the heavy to light ratios were demonstrated in the box plot of Figure 7A. Each protein and its heavy and endogenous peptide peaks were shown in Figure 7B. To obtain the robust results from the BMP2 target verification, we chose two peptides to spike in the three groups of untreated and treated autograft samples of the newly recruited set. The statistical evaluations of the target proteins are shown in Figure 7C. The target proteomic evaluations revealed that the observed expressions from the discovery data are correlated with the target SRM data analysis.

### 2.7. Western Blotting Analysis

**Figure 8:**
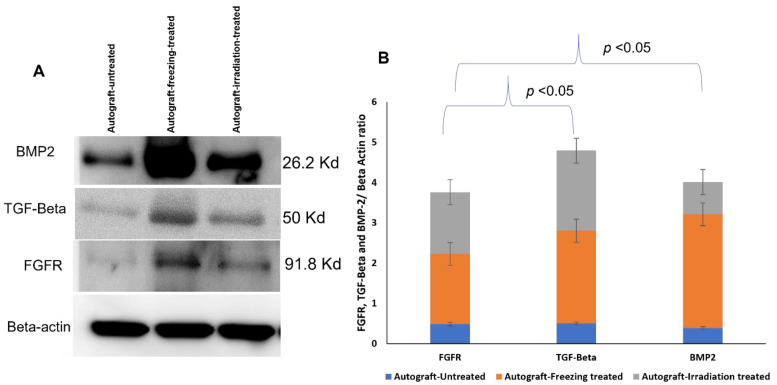
Validation of BMP2, TGF-Beta, FGFR expression levels using immunoblot analysis. (**A**) Immunoblot analysis of validating the BMP2, TGF-beta, and FGFR levels in autograft freezing treated samples compared to autograft untreated and the data was normalized using beta actin. (**B**) Bar chart showing the validation results of BMP2, TGF-beta, and FGFR comparing among treated and untreated autograft samples.

To obtain the high accuracy verification results, the expression levels of BMP2, TGF-beta, and FGFR proteins were further confirmed using Western blot. Beta-actin was loaded as a loading control and the results revealed that BMP2, TFF-beta, and FGFR showed increased expressions from the freezing-treated and irradiation-treated group compared to the untreated-autografts group. From the triplicate analysis of Western blots, the selected protein expression levels were significantly increased compared to the untreated autografts (Figure 8). The protein expression levels were illustrated in Figure 8B, which demonstrated the treatment effect and the efficacy of the autografts.

## 3. Discussion

Over the last few decades, surgery for reconstructing musculoskeletal tumors has improved greatly. New chemotherapy protocols and imaging techniques have enabled physicians to surgically remove tumors that were previously unresectable, thereby curing disease and conserving limb function. Recently, biological reconstruction techniques have gained much attention and successful outcomes in sarcoma patients. There are several techniques in limb reconstruction, such as removing the tumor-affected bone and achieving its function by mechanical implants such as prostheses or with the biologically reconstructed bone [10]. Furthermore, the biologically reconstructed bone achieves faster recovery and preserves the function of the bone [17]. Therefore, patients gain more advantages, and this technique is very helpful for the organ recovery process after the careful removal of the deadly tumor. Especially many children’s lives and limbs are being saved [10]. To implement the technique of organ replacement, there are two processes, such as using an allograft (from a donor) or an autograft (from the patient’s same bone). With the limitations of allografts and the availability of donors, it has become a challenging procedure for physicians and patients. It is also critical and important for allografts to match the patient’s size and compatibility [18]. To preserve the function of the limbs and the surrounding tissues, physicians choose biological reconstruction of bone as an ideal technique for high-grade sarcoma patients [7]. Recently, this technique showed better results than allografts in terms of bone union, remodeling, and integration. This procedure primarily uses freezing using liquid nitrogen and 1500 gamma irradiation methods to treat the tumor-bearing or diseased bone to kill the tumors resulting in the tumor-bearing bone as tumor-free. This treated bone we call autografts, and these could be inserted back into sarcoma patients for reconstruction, and it achieved faster recovery, healing, and remodeling compared to allografts in our previous studies [19,20]. In this proteomic study, we focused on the growth-factor-related proteins and their expressions in the treated and untreated autografts because they are essential for bone growth, repair, healing, and bone remodeling. From our mass spectrometry evaluations, we found that there were several protein groups that showed differential expressions from autografts treated and untreated groups. Based on gene ontology evaluations, the identified proteins were categorized as metabolic regulators, growth factors, cytoskeletal regulators, some structural and storage proteins, and so on. As our goal is to find growth factor-related and pathogenesis-related proteins, we focused on TGF-Beta family proteins and their expressions among three groups. Interestingly, BMP2, BMP7, BMP12, Osteopontin, TGF-Beta, PDGF, VEGF, and FGF proteins showed down-regulated expressions in the untreated autografts, and we compared these expressions with freezing-treated and irradiation-treated autografts to understand the treatment effect. BMP2, BMP7, and BNP12 are bone morphogenetic proteins that play an important role in the bone formation and development of cartilage [21,22,23]. Moreover, in bone remodeling, TGF-Beta plays an important role, and PDGF is a powerful bone-formation initiator [24,25,26]. On the other hand, the VEGF protein is crucial for bone repair and growth [27]. In addition to these, bone development and disease proliferation are mediated by fibroblast growth factor receptor (FGFR) proteins [28]. As a result, the down-regulated expressions of these crucial proteins may be implicated in both pathogenesis and metastasis. Consequently, the changes in these proteins may cause adverse fluctuations in bone functions as well as regulate intracellular molecules. Surprisingly, these protein expressions were increased after the treatment, demonstrating cancer cells have been killed completely and the autografts are tumor-free. We also noticed that the growth factors have been restored to their normal expressions. To further evaluate the identified results from our preliminary analysis, we selected three proteins such as BMP2, TGF-Beta, and FGFR for further validation using Western blotting and target proteomic analysis. Our validation studies successfully verified the chosen target proteins and their expressions using a newly recruited sample set. In addition to these, both the Western blotting and target SRM assay results were highly correlated with each other, which demonstrates the strength of mass spectrometry technology in validating our primary results on autografts. Furthermore, our protein–protein interaction network revealed that the identified growth factor proteins such as BMP2, BMP7, TGFB1, and collagens were tightly networked and play a key role in metabolic and intracellular signaling pathways. As a key finding, we report that TGF-beta, BMP2 and FGFR proteins resumed their expressions in the treated autografts and were involved in regulating the functions of bone union and reducing local recurrence and lung metastasis. Our results from target proteomic SRM studies that were conducted in a new set of samples provided a clearer picture of how the liquid nitrogen treated autografts are showing incredible results in resuming the expressions of proteins that helped in the regeneration of bone and its recovery.

## 4. Methods and Materials

### 4.1. Patients and Clinical Information

The current study includes a total of 24 high-grade Osteogenic Sarcoma tumor of bone (OGS) patients (male/female; 12/12; age ranging from 33 to 65 years). All the samples were obtained from Taipei Veterans General Hospital (VGH-TPE), Taiwan. With prior consent from 24 OGS patients tissue samples were freshly collected from the operation theatre after the surgery and before any treatment such as chemotherapy, radiation, and immunosuppressive medication was advised. Each of the collected specimens of OGC patients was further sectioned into three specimens and classified into three groups such as autograft-untreated, autograft-irradiation treated, and autograft-freezing treated, and utilized for comparative proteomic analysis. Table 1 shows the demographic and clinical features of the collected samples. The criteria of diagnosis for all the obtained OGS patient samples were fulfilled by a certified surgeon as well as a pathologist by the tissue biopsy examinations. The total set of samples was stored at −80 °C for further analysis. The materials and methodology conducted in this study were in accordance with the guidelines and regulations of the institutional review board (IRB) of VGH-TPE, Taiwan (IRB Approval No.2019-02-021A).

### 4.2. Extraction of Protein from Autograft Untreated and Treated Samples Preparation

The three-fold sectioned tumor tissue samples of 24 high-grade OGS patients were categorized into the following three groups: as freezing-treated (n = 24), irradiation-treated (n = 24), and untreated (n = 24). The samples of the freezing-treated group were subjected to liquid nitrogen freezing treatment for 15 min under complete sterilization conditions in order to extract the protein. The treated OGS autograft tissue samples were thawed at room temperature for 20–25 min then pulverized by mortar and pestle using liquid nitrogen. In case of irradiation-treated group, the samples were exposed to 15,000 gamma radiations to extract the protein. RIPA lysis buffer (50 mM Tris-HCl pH 7.2, 150 Mm NaCl,1% NP40, 0.1% SDS, 0.5% DOC, 1 mM PMSF, 25 mM MgCl_2_) (Sigma-Aldrich, St. Louis, MO, USA; R0278) supplemented with a phosphatase inhibitor cocktail (Thermo Fisher Scientific, Waltham, MA, USA; 78,420) was utilized for protein extraction from both the treated and the untreated autograft samples. The samples were centrifuged at 13,000× *g* for 15 min and the supernatant was separated into new tubes. The extracted proteins from all the treated autografts of both freezing and irradiation, and untreated samples were subjected to total protein concentration determination assays such as BCA and Bradford (Bio-Rad Laboratories, Hercules, CA, USA).

### 4.3. Protein Precipitation and In-Solution Digestion

Proteomic profiling of the treated and untreated samples autografts was carried out using LC-ESI-MS/MS technology. Protein samples from both groups were treated with four-fold volume of 100% ice-cold acetone and incubated overnight at −20 °C to precipitate the protein. Upon precipitation, the samples were centrifuged at 14,000× *g* for 10 min, and the pellets were dissolved in 100 µL of 25 mM NH_4_HCO_3_ with 6.5 M urea (0.1–1 µg/µL). Then, the samples were subjected to an in-solution digestion procedure illustrated elsewhere [29]. The samples were reduced using 100 mM DTT (Dithiothreitol) at 37 °C for 30–40 min followed by alkylation with 200 mM IAA (Iodoacetamide) in the dark at room temperature for 25–35 min. For the digestion of proteins, sequencing grade trypsin (Promega, Madison, WI, USA; V5111) in 50:1 ratio was used at 37 °C, and incubated overnight (16–18 h). The reaction was quenched by adding 2 µL of 50% formic acid (FA) to the protein solution and the mixture was incubated for 10 min. The digested mixture was briefly vortexed and centrifuged, and the collected supernatant was subjected to lyophilization and desalting using C18 zip-tip technique [30] to afford the desired peptide mixture.

### 4.4. Nano UPLC and Mass Spectrometry Conditions

In this proteomic study, our in-house mass spectrometry methodology conditions, which were previously demonstrated by Madda R et al. were applied successfully. As described in our earlier studies, an interface of ESI-Q-TOF MS/MS was performed to reach the full-width half maximum (FWHM) resolution at 10,000. The instrument was calibrated (@0.25 µL/min flow rate) by constantly infusing an external standard of lock mass BSA using the Nano-ACQUITY auxiliary pump with lock spray frequency intervals at 20 s. To obtain the accuracy precursor mass error was chosen as <2 ppm and the lock mass data were averaged. With a mass scan range of 50–200 *m*/*z* of 1 s we used positive V mode for all the peptide spectra that are eluted. To inject the peptides into an online nano-ACQUITY, UPLC coupled Q-TOF, Synapt-HDMS mass spectrometer (Waters Corporation, Milford, MA, USA), 400 ng peptides were digested and reconstituted in 3% ACN (Acetonitrile) and 0.1% FA (Formic Acid). Then, by using the C18 reverse-phase column (1.7 µm × 75 µm × 250 mm) (Waters Corporation, Milford, MA, USA) the digested peptides were separated. For our analysis binary solvent system contained, 99.9% water and 0.1% FA was considered as mobile phase, and 99.9% ACN and 0.1% FA performed as mobile phase B. At a flow rate of 5 µL/min using a 5 µm symmetry C18 trapping column (internal diameter 180 mm, length 20 mm) (Waters Corporation, Milford, MA, USA) with 0.1% FA was executed for all the peptides, which were primarily pre-concentrated and desalted online. Then the peptides were eluted successfully at a flow rate of 300 n/L and a gradient of 2% to 40% for 120 min into the Nano-LockSpray ion source subsequently to each injection. After all the injections the column was washed properly and equilibrated. For the comparative proteomic evaluations OGS treated with freezing, irradiation, and untreated samples were run in triplicates and the raw data was analyzed by ProteinLynx Global Server 4.2 software (PLGS: Waters Corporation, Milford, MA, USA). To obtain technical triplicates every sample was injected into the mass instrument three times such as our earlier studies [16].

### 4.5. Target Proteins Validation Using Selection Reaction Monitoring

For the target proteomic evaluations of the mass spectrometry data three proteins of interest were selected and the respective unique peptides were selected from the peptide atlas. We choose peptides with amino acid length of not more than 16 and the peptides should be unique without any missed cleavages and are not prone to post-translational modifications. The selected peptides were synthesized from the local vendor (BaChem, Taipei, Taiwan). For each peptide at least 2–3 transitions and 3 charge states were selected whose intensity is reasonably more so that it can be quantitated effectively. As a first step for the SRM assay we ran an unscheduled method to verify the crude heavy synthetic peptide signals and also to record the elution time of each peptide for every single run. Then, a retention time window of 15 min was selected for all the peptides that are selected for each protein. Then, heavy peptides were spiked into autograft samples, and were injected to Thermo Altis coupled with NanoUPLC interface with Waters C18 column and the injection volume was 2 µL. We choose a new set of samples (n = 24) for this validation to achieve a strong statistical conclusion. In order to obtain the robust quantification values, we targeted two peptides for BMP2 protein for validation of its expressions.

### 4.6. Protein Quantification

Proteomic analyses of high-grade osteosarcoma autograft tissue samples were carried out using high-resolution electron spray ionization liquid chromatography and tandem mass spectrometry (LC-ESI-MS/MS) analysis. The proteins identified from LC-ESI-MS/MS investigation were quantified by label-free quantification using PEAKS Studio X (Bioinformatics Solutions Inc., Waterloo, ON, USA) [31,32]. Analyzed triplicate independent samples were compared among freezing-treated, irradiation-treated, and untreated/control groups of high-grade OGS autograft patients. The raw data files of the analyzed samples were imported from the mass spectrometry instrument and uploaded to the quantitative PEAKS software program. The identified proteins from the triplicate tested samples each spectrum and its interpretation along with the alignment of the ion chromatogram and retention times were studied. To achieve better accuracy, the retention times were set in the range from 600 to 10,500 s. The identification of proteins from the raw data was accomplished in the same manner as described in our earlier study [16,33]; an Uniprot’s reference database of Homo sapiens (release 03_2014) [34] contained 20,272 entries were added and combined with a decoy database (the sequences were reversed) was used. For label-free quantification a set of parameters were specified as follows: digestion by trypsin, with 2 missed cleavages; precursor mass tolerance was 10 ppm; fragment mass tolerance: 0.7 Da, minimum charge: 2, maximum charge: 3, carbamidomethylation, oxidation (M), and deamidated (N and Q) were specified as fixed and variable modifications. The false-positive identification rate was determined by employing the estimated spectra against the decoy database. A false discovery rate (FDR) of <1%, with a peptide score of −10 log *p* ≥ 20 was executed to obtain precise identifications of the proteins from each sample.

Moreover, the relative protein and peptide abundance in the tested samples was determined using the peptide feature-based quantification as described in our earlier studies. For the accurate identification of peptide intensity differences among two samples the peptide signal intensity is equivalent to the abundance of the peptides in the sample, thus the peptide features were corresponding accurately. The differences in peptide intensity among autograft treated and untreated samples were quantified efficiently using these parameters. The extracted ion chromatograms (XICs) and the area under the curves (AUC) were measured and compared among the three analyzed runs. The total cumulative peak area of the identified proteins was determined by choosing only the unique peptides that are specifically stipulated to the particular proteins that were chosen. As mentioned in the earlier studies, Ref. [33] based on the target/decoy database FDR was calculated. Considering the chance of obtaining one false positive in 20 observations the peptides with FDR >1% were chosen as true positive hits. With this active feature-based quantitative approach the identified peptides with *p*-values between < 0.05 and 0.01 that were identified in at least three observations from the OGS both the treated and untreated were compared and measured. To determine the significance of protein expressions between treated OGS and untreated samples was explained in statistical analysis section. The obtained spectral datasets were quantified and normalized to obtain the abundance factor values (triplicate analysis of the LC-MS/MS were averaged). Among the treated and untreated groups of high-grade osteosarcoma autograft samples, the differentially expressed proteins (DEPs) were identified and generated in a heatmap by peaks X software, which illustrates the protein expressions. To limit the false positives, an individual false detection (FDR) rate was applied and the proteins with *p* > 0.05 were excluded from further analysis. Moreover, proteins, which were identified only in one of the three technical replicates, and with an XIC value lower than 100,000 were considered as absent (noise) and omitted from further study. Both the treated and untreated OGS sample’s technical replicates XIC values were averaged and quantified, and the ratios of OGS-untreated/OGS-treated with freezing and irradiation were employed to identify the differentially expressed proteins as down-regulated proteins with <0.3–0.5-folds. Upregulated proteins were denoted with OGS-untreated/OGS-treated with a fold change from <1.5 to 2.

### 4.7. Protein Identification

The protein identification analysis was further carried out on the altered proteins from this study using Mascot Software (Matrix Science version 2.2, http://www.matrixscience.com, accessed on 22 August 2021) [35] search engine along with the UniProtKB database (UniProt release 2015-10) [34,36] and National Center for Biotechnology non-redundant (NCBInr). The following options were chosen to screen the proteins precisely; digestion by trypsin with two missed cleavages while setting the carbamidomethyl and oxidation (M) as constant modification and variable modification, respectively. Mass tolerance of 50 ppm and 0.1 Da MS/MS were specified. FDR of <1% were selected to eliminate the false identifications from the obtained data. The proteins that are consistently identified based on the stated parameters from all the three technical replicates or at least two of the three analyses were selected for further evaluations. The Mascot database was employed to determine the theoretical molecular mass (MW) and isoelectric point (pI) of the identified proteins.

### 4.8. Bioinformatics Analysis

Database such as gene ontology (GO) (http://www.geneontology.org/, accessed on 22 August 2021), PANTHER version 7.1, and the DAVID (http://david.abcc.ncifcrf.gov/, accessed on 22 August 2021) (Database Annotation Visualization, and Integrated Discovery) [37,38] for functional analysis were performed to understand the involvement of identified proteins in biological processes (BPs) and their molecular functions (MFs), along with the protein categories and cellular components (CCs). Furthermore, the identified proteins from high-grade OGS autograft-untreated vs. treated were evaluated for any protein–protein interactions (PPI) by analyzing the results using STRING (Search Tool for the Retrieval of Interacting Genes/Proteins, Version 9.1) PPI networks ( http://string-db.org/, accessed on 22 August 2021) and specified the high score of 0.09 along with the default parameters for the significant results. Our analysis resulted in a better understanding of the identified proteins and their biological context, and involvement in various pathways that are playing a potential role in pathogenesis and diagnosis of chondrosarcoma.

### 4.9. Statistical Analysis

Statistical analysis was carried out to confirm the variations in the percentage of volume and relative intensity of the protein profiles from the triplicate analysis of high-grade OGS autograft-untreated vs. freezing and irradiation treated patients’ samples. The spectral counting evaluations were employed in order to understand the altered expressions of the proteins quantified using LC-ESI-MS/MS data. Three technical replicates were processed for every single sample and the average of the obtained abundance spectra was calculated. The data are expressed as mean ± standard deviation (SD), which was determined using analysis of variance (ANOVA) assessment [39], and Mann-Whitney U-test was performed by SPSS statistical package (SPSS19, SPSS Ltd., Woking, Surrey, UK) for Windows. The probability values <0.05 and <0.01 were considered as statistically significant and highly significant, respectively.

### 4.10. Western Blot Analysis

The selected proteins were validated using Western blotting analysis in a new set (n = 12) of high-grade OGS autograft bone tissue samples and grouped as post and pre-treated specimens. SDS-PAGE was employed to separate the proteins onto an electrotransferred PVDF membrane (Millipore Corporation, Bedford, MA, USA) at 100 V for 60 min. Then, the transferred protein membranes were immersed in 5% non-fat milk in a TTBS solution [0.2 M TRIS-HCl (pH 7.6), 1.37 M NaCl, 0.1%Tween-20] [40], for 1 h at room temperature. Further, the proteins were incubated with primary antibodies, FGFR rabbit monoclonal antibody (catalog no. ab76464, 1:1000 dilution), protein TGF-beta rabbit mAb (catalog: ab92486, 1:1000 dilution), BMP2 rabbit monoclonal antibody (catalog no. ab14933, 1:1000 dilution), and beta-actin rabbit mAb (catalog no. ab8227, 1:1000 dilution) for overnight at 4 °C. The utilized antibodies were purchased from Abcam (www.abcam.com, accessed on 22 August 2021) (Cambridge, UK). Furthermore, the membranes were washed and incubated in 5% non-fat milk in a TTBS solution for 3 h at room temperature followed by rinsing trice in a TTBS solution for 5 min each. Then incubated 1 h at room temperature with a horseradish peroxidase-conjugated goat anti-rabbit antibody (Zhongshan Golden Bridge Biotechnology Co., Ltd., Beijing, China; catalog no. 7074), and washed 3 times for 5 min rinses in a TTBS solution. The blot was developed with a Super ECL Plus kit (Applygen, Beijing, China), and the signal was exposed to an X-ray film. Upon scanning the images, the intensity of each band was captured using an Image Master 2D Platinum version 5.0 (GE Healthcare Amersham Bioscience, Amersham, UK). Then, the intensity of each band that consistently observed was standardized as a percentage of the total intensity. Additionally, the relative expression abundance of the identified proteins in the tested samples was referred to a relative volume. Then, each band intensity that was consistently observed was standardized as a percentage of the total intensity, and as referred to a relative volume that represents the relative expression abundance of the identified proteins in the tested samples. The protein expression stability was evaluated using the relative expression abundance.

## 5. Conclusions

In conclusion, it is a key finding of our study that tumor-bearing bones that are treated using liquid nitrogen/LN-freezing-treated and irradiation-treated are excellent tools for the biological reconstruction technique for high-grade sarcoma patients. As such, these findings provide an insight into potential pathways that play a huge role in recovery and reducing recurrence and metastasis. The current study has a large representation of high-grade osteosarcoma patients who have chosen biological reconstruction as their treatment approach. With the advent of an increasing number of failed treatments for osteosarcoma and losing limbs, our proteomic study may inform clinical research input in demonstrating the treatment effect and their success in using autografts in high-grade osteosarcoma patients.

## Figures and Tables

**Table 1:** Patient demographic information.

Classification	Irradiation-Treated (n = 24)	Freezing-Treated (n = 24)
Gender		
Male	12	12
Female	12	12
Age (mean)	40.5 ± 4.2	40.5 ± 6.1
Tumor Location		
Distal femur	4	4
Proximal femur	2	2
Proximal tibia	4	4
Proximal humerus	2	2
Tumor Length (mean)	10.5 ± 6.2	11.2 ± 5.8
Follow-up (mean, months)	55.78 ± 13.6	55.96 ± 13.2

## Supplementary Materials

The following supporting information can be downloaded at: https://www.mdpi.com/article/10.3390/ijms23137409/s1.
