# Supplementary material for: Analyzing BMP2, FGFR, and TGF Beta Expressions in High-Grade Osteosarcoma Untreated and Treated Autografts Using Proteomic Analysis
Source: Int J Mol Sci. 2022 Jul 3;23(13):7409. doi: 10.3390/ijms23137409 (PMC9266757; doi:10.3390/ijms23137409)
Supplement: Supplementary file 1 [file ijms-23-07409-s001.zip › ijms-1755216-supplementary.pdf]

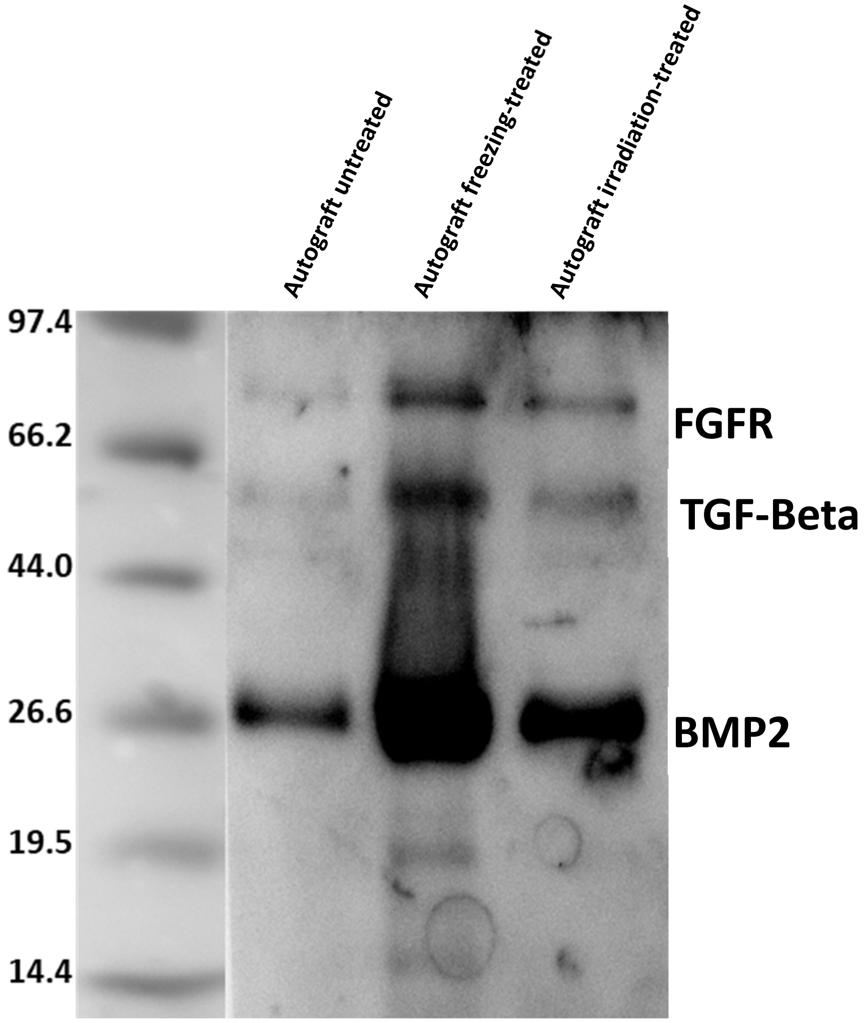

Supplementary Figure S1. Uncropped western blot images containing molecular weight markers of Autograft-untreated and Freezing treated and Irradiation-treated validating proteins of FGFR, TGF-beta and BMP2 are shown.
